# Conducting interactive experiments online

**DOI:** 10.1007/s10683-017-9527-2

**Published:** 2017-05-09

**Authors:** Antonio A. Arechar, Simon Gächter, Lucas Molleman

**Affiliations:** 1 Department of Psychology, Yale University, New Haven, CT USA; 2 CeDEx, University of Nottingham, University Park, Nottingham, NG7 2RD UK; 3 School of Economics, University of Nottingham, University Park, Nottingham, NG7 2RD UK; 4 CESifo, Schackstrasse 4, 80539 Munich, Germany; 5 IZA, Schaumburg-Lippe-Strasse 5-9, 53113 Bonn, Germany; 6 Max Planck Institute for Human Development, Lentzeallee 94, 14195 Berlin, Germany

**Keywords:** Experimental methodology, Behavioral research, Internet experiments, Amazon Mechanical Turk, Public goods game, Punishment, C71, C88, C90, D71

## Abstract

Online labor markets provide new opportunities for behavioral research, but conducting economic experiments online raises important methodological challenges. This particularly holds for interactive designs. In this paper, we provide a methodological discussion of the similarities and differences between interactive experiments conducted in the laboratory and online. To this end, we conduct a repeated public goods experiment with and without punishment using samples from the laboratory and the online platform Amazon Mechanical Turk. We chose to replicate this experiment because it is long and logistically complex. It therefore provides a good case study for discussing the methodological and practical challenges of online interactive experimentation. We find that basic behavioral patterns of cooperation and punishment in the laboratory are replicable online. The most important challenge of online interactive experiments is participant dropout. We discuss measures for reducing dropout and show that, for our case study, dropouts are exogenous to the experiment. We conclude that data quality for interactive experiments via the Internet is adequate and reliable, making online interactive experimentation a potentially valuable complement to laboratory studies.

**Electronic supplementary material:**

The online version of this article (doi:10.1007/s10683-017-9527-2) contains supplementary material, which is available to authorized users.

## Introduction

Online labor markets such as Amazon Mechanical Turk (MTurk) are increasingly popular tools for behavioral scientists. With their large and diverse pools of people ready to promptly perform tasks for pay, these markets present researchers with new opportunities to recruit participants for experiments.^1^ Studies from across the social sciences have systematically compared data collected online with data from the physical laboratory. Their conclusions are promising: classic results from psychology and economics have been replicated using online samples, and the data obtained online is deemed as reliable as that obtained via traditional methods.^2^

Despite its great potential, behavioral research online has so far remained largely limited to *non*-*interactive* decision-making tasks or one-shot games with simultaneous decisions. Current online studies of social behavior often use survey software such as *Qualtrics* or *SurveyMonkey* to document decision making in tasks that participants complete individually, and emulate interactions through post hoc matching. Although this approach can be powerful, it does not permit the study of repeated, ‘hot’ interactions where live feedback between participants is essential. Experimental designs with live interaction are rarely implemented online, partly because there is not yet a widely-used web-based equivalent of z-Tree (Fischbacher 2007).^3^

In this paper, we assess the potential for *interactive* online experiments, where a set of participants interacts for more than one repetition. Interactive experiments raise novel challenges throughout the whole life cycle of an experiment. Our approach is to discuss these challenges, that is, methodological differences and similarities between interactive experiments in physical and online laboratories. We discuss these step-by-step, from recruitment to dismissal of participants after the experiment.

A particularly important challenge of interactive online experiments relates to participant dropout. While in the physical laboratory participants rarely leave a session, online experiments are more prone to dropouts which affect both the participant who is dropping out and their interaction partners (who still have to be paid for their participation). If dropouts happen for reasons exogenous to the experiment—e.g. due to network problems, frozen screens, or random distractions—they are just a (costly) nuisance to the experimentalist. Much more problematic are dropouts that happen *endogenously*, that is, people quitting because of what has happened in the experiment. Such dropouts could jeopardize the internal validity of experiments (Zhou and Fishbach 2016).

As a case study we replicate a repeated public goods game with and without peer punishment used in cross-cultural research (Herrmann et al. 2008), employing a sample of US participants recruited via MTurk.^4^ We chose to replicate this experiment because it is fairly long and logistically complex. It is a within-subjects design with two experimental conditions of ten periods each, where, after the first set of ten periods, participants receive new instructions. Moreover, this experiment has often been replicated, and its design allows us to evaluate whether dropouts depend on the experimental conditions (that is, the presence or absence of punishment). We report data from participants recruited via MTurk (62 groups) and participants from the physical laboratory (18 groups). We used our own software LIONESS (Sect. 2.5), developed for conducting interactive online experiments.

We observe that basic patterns of behavior online are similar to those in the laboratory. In the absence of punishment, aggregate levels of cooperation are higher on MTurk than in the laboratory, but show similar rates of decay over time. Moreover, our econometric analysis reveals that in both of our samples the group contributions strongly determine the level of cooperation. The introduction of punishment promotes the emergence and maintenance of cooperation in both samples. Punishment is mainly prosocial in nature in both samples (cooperators punish non-cooperators) but occurs less frequently online.

Our most important result is that, in our implementation, dropouts are most likely due to reasons that are exogenous to the experiment. Together with the replication of findings from the laboratory, our results suggest that online interactive experiments can be a reliable tool for collecting internally-valid data and hence are a potentially valuable complement to the physical laboratory.

Our paper contributes to a recently-emerged literature on the reliability of data gathered on online labor markets such as MTurk (see references in footnotes 1–3). The most important predecessor of our paper is Anderhub et al. (2001), who compared online and laboratory experiments in the very early days of experimentation on the Internet. They also provide a methodological discussion that, however, could not consider the specific properties of modern online labor markets where the bulk of present-day online experimentation is happening.^5^

The remainder of this paper is structured as follows. In Sect. 2, we introduce the experimental design. In Sect. 3, we discuss the conceptual and logistical differences between conducting interactive experiments in the laboratory and online and lay out our approach for dealing with them, highlighting important aspects of the data-collection process relating to attention and attrition. Section 4 shows the results of our experiment, systematically comparing cooperation and punishment behavior in our two samples. In Sect. 5 we present a detailed analysis of attrition in our online experiment. Finally, in Sect. 6 we make concluding remarks.

## A case study to compare online and laboratory experiments

We base our discussion of online and laboratory experiments on a well-established paradigm: a public goods game with and without punishment (Fehr and Gächter 2000, 2002). In this section, we present the design of our experiments conducted in the laboratory and replicated online with a sample of participants recruited via MTurk. For the laboratory and the online samples, instructions and experimental screens were identical (screenshots are presented in the Online Appendix A).

### General setup

Our experiment follows the within-subject design of Herrmann et al. (2008) and implements a repeated four-person public goods game with two conditions: one without punishment followed by one with punishment. Groups were constant throughout the experiment (‘partner matching’), and each condition ran for ten periods. Participants were aware that there were two ‘parts’ to the session (which corresponded to the conditions without and with punishment) but learned about the details of the second part only after the first one had finished.

At the beginning of a session, participants read on-screen instructions for the first experimental condition: the public goods game without punishment. Experimental instructions were shorter than those in Herrmann et al. (2008) (see Sect. 3 for rationale). Participants could start the interaction phase only once they had completed a set of comprehension questions.

### Condition 1: a public goods game without peer punishment

In each period of the 10-period game, all four group members received an endowment of 20 points and simultaneously decided how many of those points to keep for themselves, and how many to contribute to a ‘group project’ (i.e. the public good). After all members had made their decision, the sum of all contributions was multiplied by 1.6 and distributed equally among all group members irrespective of their contributions. This setup reflects a social dilemma: in each period overall earnings are highest when each of the group members contributes all 20 of their points to the public good, while individuals maximize their earnings by contributing 0 regardless of the contributions of the others. Once all contributions had been made participants learned the result of that period. Apart from their own contribution and earnings, they were informed of the average contribution in their group. Subsequently, a separate screen showed the contributions of each of their fellow group members.

### Condition 2: a public goods game with peer punishment

Once the 10 periods of Condition 1 were over, participants received new on-screen instructions about Condition 2. This condition also consisted of ten periods and was completed in the same groups as Condition 1. Again, the periods started once all group members had completed the comprehension questions. The decision situation was like Condition 1, but we introduced one change: once participants learned the contributions of each of their group members, they could assign up to 10 deduction points to each of their peers. Each assigned deduction point resulted in a loss of 1 point for the participant assigning it, and a loss of 3 points for its target. At the end of each period a separate screen informed participants of the total number of points they assigned and received. In cases where a participant made a loss during a period, only the costs of assigning deduction points would count towards the final earnings (cf. Herrmann et al. 2008). Each session concluded with a questionnaire including demographic items.

### Online and laboratory sample

In all sessions, participants received instructions and made their decisions via web browsers. The program was implemented in the experimental software LIONESS (Sect. 2.5). Both online and in the laboratory, sessions took 28 min on average. This is considerably shorter than the original study by Herrmann et al. (2008), but longer than typical tasks on MTurk.

For our *online* sample, we recruited participants via MTurk, restricting their geographical location to the USA (for comparability with our laboratory sample, see below). Results are based on 24 sessions, with 248 participants in total (62 groups of four). The average age of participants in this sample was 31.5 years (s.d. 9.06), and 38.4% were female. Average earnings in our online sample were $6.69 (s.d. $1.03), which were paid via MTurk.

The data from our *laboratory* sample were collected at universities in two different cities in the USA (Harvard University, Boston MA; and Yale University, New Haven CT) over 8 sessions, with 72 participants in total (18 groups). Laboratory participants were invited through e-mails using the online recruitment software SONA. The average age for participants in this sample was 25.2 years (s.d. 7.45) and 42.3% were female. Average earnings in our laboratory sample were $20.02 (s.d. $1.65), paid in cash upon session completion. To conform to standards of the respective laboratories and average expected wages on MTurk, we used an exchange rate of $0.02 in the laboratory and $0.01 in our online sessions; show-up fees were $10 and $1 in the laboratory and online, respectively.^6^

### The software used to conduct interactive experiments: LIONESS

We conducted both the laboratory and online experiments with LIONESS (Live Interactive Online Experimental Server Software). LIONESS provides a basic architecture for conducting interactive experiments online. Its key features reflect the solutions to the logistical challenges discussed in Sect. 3; dynamically grouping participants to minimize waiting times, regulating interactions in groups, promoting participants’ attention to the experiment and dealing with participants dropping out of an experiment. The software developed for the experimental conditions reported here can be downloaded at: http://lioness.nottingham.ac.uk.

## Methodological differences in conducting interactive experiments in the laboratory and online

### The online laboratory MTurk

While our discussion of online experiments is based on an MTurk sample, many issues also hold for other online platforms (cf. footnote 1). MTurk is a large online labor market, which offers an active pool of over 500,000 workers. The MTurk workforce completes over 40,000 Human Intelligence Tasks (HITs) every day (www.mturk-tracker.com; Difallah et al. 2015; Ipeirotis 2010). MTurk ‘workers’ browse HITs that are published by ‘requesters’ who provide a brief description of the task, its expected duration and the minimum payment workers will receive upon completion (see Online Appendix A for screenshots of the HIT as published on MTurk). HITs typically involve short individual assignments which computers are currently unable to perform (Berinsky et al. 2012), such as the processing of images or data cleaning. Due to the sheer size of the pool of workers ready to perform tasks for pay, MTurk enables researchers to conduct large-scale experiments and to implement an effective random assignment of participants to different conditions beyond the capacity of a typical physical laboratory.^7^ While not primarily designed for academic research, MTurk has the potential for efficient data collection. As mentioned in the introduction, questionnaire studies and experiments without repetitions (e.g. one-shot Prisoner’s dilemma) conducted with MTurk participants have produced results comparable to those obtained from laboratory samples (e.g. Paolacci et al. 2010; Horton et al. 2011; Goodman et al. 2013).

Despite their promise, online behavioral experiments have conceptual and logistical challenges that are usually not present in the laboratory. Here we focus on differences between laboratory and online experimentation that are specific to *interactive* designs. See Buhrmester et al. (2011) and Paolacci and Chandler (2014) for extensive discussions of differences regarding non-interactive (survey-style) designs.

### A step-by-step comparison of laboratory and online experiments

We now discuss the implementation of interactive experiments in the laboratory and online via MTurk, based on the design we presented above. Our discussion is chronological in the way a typical experiment proceeds from recruitment to dismissal of participants. The four following subsections discuss the main phases of a typical experimental session (recruitment, session start-up, interactive decision making and payment). Along the way, we highlight the extent to which our approach bridges these gaps in the (relatively long and logistically-challenging) experiment presented above. Table 1 provides a concise overview of the issues discussed in this section.

**Table 1:** Methodological differences in conducting interactive experiments in the laboratory and on MTurk

Phase/challenge	Laboratory	Online (MTurk)
*Recruitment*		
Show-up fees	Typically a small part of total payoffs. Guaranteed when participant shows up to the session	Relatively large show-up fees promote recruitment rates, thereby facilitating prompt group formation. Experimenter can approve or reject the task submitted; if rejected no fee is paid
Inviting participants	Invitations sent well in advance, participants commit to a session. Recruitment often from a pre-existing database	Sessions advertised online as HITs and can be completed immediately
Selection into the experiment	At sign-up, participants know very little about the experiment. Details of the task are communicated once participants are in the laboratory	Experiments are typically advertised as HITs with a brief task description. ‘Workers’ browse available HITs and accept those of their preference
Experienced participants	Invitation conditioned on well-defined criteria of the laboratory’s records	HITs targeted at subsets of MTurk workers; experimenter can specify exclusion criteria. Many MTurk workers will have participated in many prior studies
*Session start*-*up*		
Duplicate participants	Registration protocols usually prevent duplicate participation	Amazon acts against multiple worker accounts, but they exist
Comprehension	Participants can ask questions; comprehension questions ensure understanding	Experimenter is physically absent and cannot answer questions directly. Compulsory comprehension questions can be added but may make experiment (too) long for some participants
*Experimental interactions*		
Forming groups	Easy to guess how many participants will attend; group settings can be pre-defined	Hard to guess how many participants will attend; groups can be constructed ‘on the fly’
Deception	In experimental economics deception is prohibited and laboratories foster reputations for non-deception	Because all requesters use the same subject pool, some participants may have experienced deception because requesters from other disciplines may use it
Communication	Hardly an issue; experimenter can restrict communication between subjects	Participants may in principle collude through external channels though this is difficult in practice
Experimental flow	Closed form software like z-Tree specifies session progress	Scripted browser navigation specifies progress
Attrition (‘dropout’)	Hardly an issue; participants that start a session usually finish it	Major challenge to internal validity, if dropout rates vary with treatment, selection bias may arise
*Payment*		
Payments	Cash usually paid upon completion	Automatic transfer through Amazon
Cost per participant	Relatively high but predictable	Relatively low but varies with attrition

#### Recruitment

In a typical laboratory experiment, participants receive a show-up fee for attending. Still, the main part of the participant’s payment is usually determined by the decisions made over the course of the session. In a typical task on MTurk, participants are paid a flat reward per HIT, and the part of the earnings determined by their decisions can be added to their payments as a ‘bonus’. Consequently, a HIT that pays a relatively large flat fee usually draws more attention than one that promises a large bonus. This is particularly relevant for interactive experiments where participants need to wait for others to form a group at the start of a session (see below).

Sessions in the laboratory are pre-scheduled. A database contains the contact details of a pool of aspiring participants, who can register (and cancel) within a determined time window. Pre-scheduling ensures that the number of participants can be anticipated quite accurately before a session takes place, and including a small number of backup participants can prevent problems associated with unannounced non-attendance. Online platforms such as MTurk allow for instant recruitment of participants, facilitating a time-efficient method of data collection.^8^

To take advantage of these opportunities, LIONESS was ready to accommodate new participants during a time window specified by us, while capping the maximum number of entrants. In our experiment, we invited participants to sign up within 20 min of the HIT being posted and allotted them 45 min to complete the task. In addition, we asked them to start immediately. Recruitment rates were high (in a typical session with 100 slots, the first 50 participants normally entered within the first 5 min after the publication of the HIT), facilitating prompt group formation once participants had read and understood the instructions.^9^

Invitations to laboratory experiments rarely reveal any information on the contents of the experiment. On MTurk, however, participants browse various tasks that are currently available to complete for pay. This requires a HIT description giving the workers some idea of what the task will involve. To avoid self-selection (based on the topic of the experiment) into interactive experiments as much as possible, an experimenter can leave out any detailed information in the HIT description.^10^ For example, we did not announce that these are public goods experiments. On the other hand, it is essential that workers know that the HIT will involve live interactions with other people, and therefore they are expected to complete the interactive HIT without delay and without interruptions. The HIT description within MTurk is an appropriate place to make participants aware of this (see Online Appendix A for screenshots).

Typical laboratory subject pools are replenished annually when a new cohort of first-year students arrives on campus, and recruitment software allows invitation of only those participants with no (or little) experience with the experimental paradigm of a study. By contrast, the pool of MTurk workers (MTurkers) is replenished more regularly, but oftentimes MTurkers quickly acquire a sizable experience participating in hundreds of academic studies of all kinds.^11^ While researchers may have reason to believe that (frequent) prior experience could be an issue for their experiment (for a discussion of “lab rats” see Guillen and Veszteg 2012), MTurk facilitates inviting participants based on various criteria (e.g. number of HITs completed, their success rate or their geographic location). In addition, post-experimental questionnaires can include self-reported measures of participants’ familiarity with decision-making experiments and specific experimental paradigms.

In our case, we used MTurk’s options to restrict the geographical location of the participants to the United States for comparability with our laboratory sample. In addition, to increase the likelihood that participants completed our HIT with care, we only allowed workers with at least 90% of their previous HITs approved by requesters to participate (see Peer et al. 2014 for a detailed discussion of approval rates).

#### Session start-up

For many studies, it is essential that participants take part only once. In the laboratory it is relatively straightforward to implement this, particularly if the experimenter uses recruitment software such as *ORSEE* (Greiner 2015), *SONA* or *hroot* (Bock et al. 2014) and is physically present during laboratory sessions to confirm identities. For online sessions, however, re-takers may seriously compromise the data (e.g. by operating two browsers within the same experiment, potentially even controlling two players within the same group). Accordingly detecting them requires specific measures. Within a session, we prevented duplicate participation by logging the user’s IP address and blocking users that had already been connected to the experimental server. Between sessions, we used third-party software to prevent workers who had already participated in a specific HIT from being invited for future sessions.^12^

In a typical laboratory session participants can ask questions which the experimenter can answer in private, before the interactive phase of the experiment begins. For online sessions this is not feasible. To ensure that participants had a thorough understanding of the experimental decision situation and did not rush through the instructions, we introduced compulsory comprehension questions which participants had to solve before entering the decision-making phase of the experiment.^13^ A fraction of participants who entered the experimental pages did not proceed past the instructions and never reached the comprehension questions. In our online sample, 83.2% of the individuals who did reach the comprehension questions solved them successfully.^14^

#### Experimental interaction

In the laboratory, all participants typically arrive at a session at the same time and will simultaneously complete comprehension questions. By contrast, participants in online sessions may arrive during a time window set by the experimenter (20 min in our case), and the timing of completing comprehension questions may therefore vary substantially. Accordingly, we formed groups ‘on the fly’: participants who successfully completed the comprehension questions waited in a ‘lobby’. As soon as this lobby contained sufficient participants, a group was formed and its members were sent to the interaction phase of the experiment. An alternative (yet considerably less time-efficient) approach is to run pre-tests with participants and to build a ‘standing panel’ from which candidates for experimental sessions are recruited (see Suri and Watts 2011; Gallo and Yan 2015).

Although deception is uncommon in experimental economics, participants on MTurk are likely to encounter studies using deception (e.g. Pfattheicher and Schindler 2015). Participants may therefore be skeptical about the truthfulness of experimental instructions and doubt if their interaction partners are real people and not robots pre-programmed by the experimenter publishing the HIT. To promote trust between us (as experimenters) and the participants, we continuously strive to maintain a good reputation on our MTurk requester account (our records and those of other requesters can be found at https://turkopticon.ucsd.edu). In addition, our HIT description stated explicitly that groups were formed of real people recruited from MTurk.

To keep the attention of the participants focused on the experiment (and not have them dropping out in the very first period of the game), we clearly communicated the number of other participants they were waiting for at any given moment, and we added an on-screen countdown indicating the maximum amount of time left before participants could choose to leave the experiment if no group could be formed. When this timer reached zero in our experiment, participants could choose to either return to the lobby and wait for two additional minutes or to leave the session and collect their participation fee (of $1). This procedure led to a total of 89% of participants who correctly completed the comprehension questions being successfully matched into a group and starting the interaction phase.^15^ The remaining 11% could not be matched in a group of four, and were paid their participation fee.

While in the laboratory the experimenter can monitor and enforce any restriction of communication between participants, it is in principle harder to categorically exclude the possibility that online participants communicate with their interaction partners through external channels. We ran relatively large sessions in which participants could not be identified to prevent them from colluding via online forums such as *Reddit* or *MTurk Crowd*. In fact, forum discussions, which are usually moderated and prohibit the dissemination of the content of HITs and the discussion of strategies, typically center upon the attractiveness of a HIT in terms of earnings and length rather than its content (Chandler et al. 2014). Therefore, communication between participants is a potential problem for online experiments, but it is not any more severe for interactive designs. A similar argument could be made for communication with other people who are not participating in the task at hand (e.g. someone else in the room while completing the task). Although such communication is harder to control in online experiments than in the physical laboratory, this issue is not specific to interactive designs either.

The most severe problem for online interactive studies, and the largest discrepancy with laboratory experiments, is *attrition* (participant dropout). In laboratory sessions participants very rarely leave or turn out to be unable to complete a session. In online experiments, attrition is a major issue—there is no straightforward way to prevent participants from leaving a session by closing their browser window or failing to submit responses to experimental decision situations due to technical problems. Moreover, in contrast to the laboratory, interaction partners are geographically scattered and the progress of an experiment depends on their joint attention to it. Typically, groups proceed at the pace of the slowest participant and long waiting times increase the risk of reduced attention, which may ‘cascade’ through the group. Thus, we took measures to retain attention and promote successful completion.^16^ In our case, we used on-screen timers and told participants that failure to reach a decision in due time would result in their removal from the experiment without payment. Furthermore, in the event of a group member dropping out we notified the remaining participants of that and they continued in their reduced group.^17^ Data from incomplete groups is not included in the results reported in Sect. 4. An alternative approach to dealing with dropouts is to terminate the whole group once a member drops out. This may, however, damage the reputation of the experimenter as participants will be unable to earn as much as they had anticipated.

Our procedure of letting smaller groups continue ensures that real people generate all the information that participants respond to. Alternative solutions to non-responding participants, such as introducing random decisions or repeating previous decisions (e.g. Suri and Watts 2011; Wang et al. 2012), may affect the behavior of those who are still in the experiment (now responding to partially computer-generated information) which potentially compromises the internal validity of the data from groups affected by a dropout. It also raises issues of deception if such computer-generated information is not disclosed. Moreover, this procedure may also compromise the validity of data from groups unaffected by attrition, as participants cannot know whether their interaction partners’ behavior shown to them is real or generated by a computer.

Due to the nature of conducting research via the Internet, some level of attrition seems unavoidable. Attrition rates are likely to vary with factors such as group size, complexity of the decision situation, and the pace of the experiment.^18^ Despite our measures to prevent attrition, 84 participants (18%) who started the interaction phase dropped out at some point in our experiment.^19^ As these participants were distributed across experimental groups, the fraction of the data set affected by these dropouts was considerably larger.

**Fig. 1:**
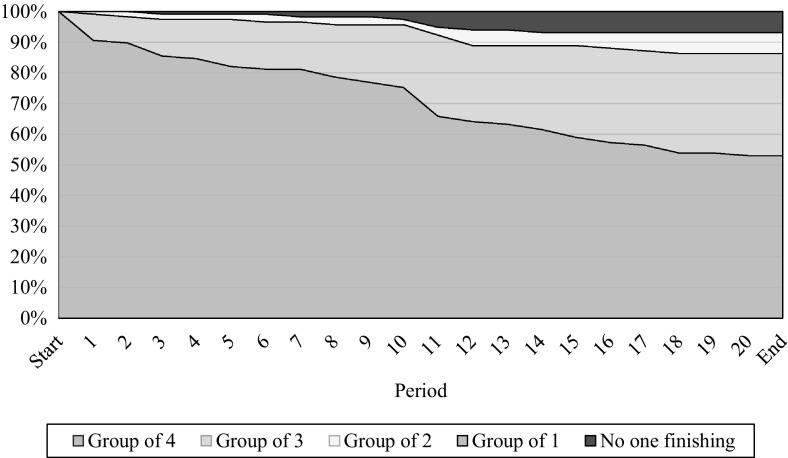
Attrition throughout the course of the experiment. *Colors* depict the group size. We always started with groups of four but let participants continue if a member dropped out. (Color figure online)

Figure 1 tracks the distribution of group sizes over time. All groups are initially formed of four group members, but a group’s size may decrease over the course of the experiment as participants drop out. The figure shows that our experiment suffered from quite substantial attrition and only 53% of the groups finished with all four members. Loss of group members was particularly likely around the ‘waiting room’ stages preceding periods 1 and 11. Specifically, in period 1 dropouts are presumably increased due to participants losing attention while waiting for their group to form. Similarly, before period 11 started participants had to wait until each of their group members had completed the comprehension questions, which could take a considerable amount of time. In some cases, this led to the termination of the whole group.

#### Payment and costs of experiments

After a session is over, participants are typically paid according to their performance. MTurk, like other crowdsourcing platforms (see footnote 1), facilitates secure payments. The experimental software can generate a random code for each participant which can be matched with their MTurk ID, allowing for payments according to performance in the experiment. It is important to process payments immediately to maintain a good reputation as an MTurk requester.

All in all, typical costs per useable data point in an experiment with participants recruited via MTurk are likely to be lower than in a laboratory experiment. Nevertheless, these costs may vary with attrition rates, which can be affected by the specific features of the experiment such as its length and group size (as one dropout may compromise the data of the whole group). In our case, laboratory participants earned $20.02 on average. Therefore, with a group of four as the unit of observation, a useable data point cost us $80.08. Corresponding costs online were $47.32.^20^

## Results

### Contribution behavior

**Fig. 2:**
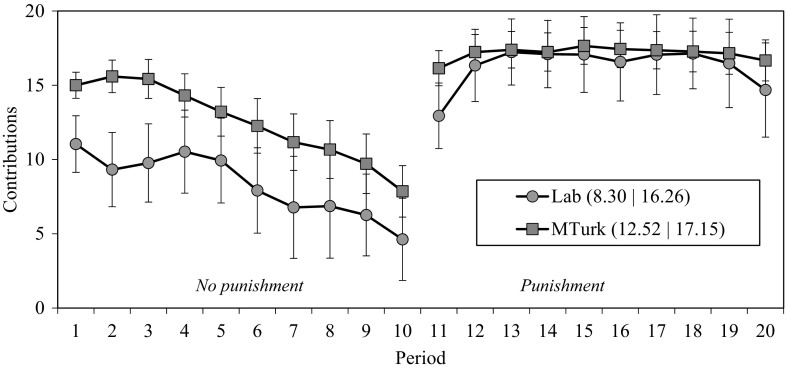
Contributions over time.* Numbers in parentheses* are the mean contributions in each experimental condition. *Error bars* indicate 95% confidence intervals (clustered at the group level)

Figure 2 shows the aggregate contribution dynamics for both the online and laboratory samples. Only the data from groups with no dropouts are reported. In the condition without punishment, overall contributions were higher in our online sample than in the laboratory (12.52 vs 8.30, *p* = 0.003).^21^ This result is consistent with recent literature reporting college students to be less cooperative than non-students and other adults (Belot et al. 2010; Carpenter and Seki 2011; Gächter et al. 2004; Stoop et al. 2012). The difference in contributions emerges right in the very first period of the game, with online participants contributing substantially more to the public good (15.00 vs 11.04, *p* = 0.001). Higher contributions by MTurkers can be only partially explained by the higher average age in the MTurk sample (OLS fitted to contribution decisions in the first period of the first condition: age *β* = 0.132, *p* = 0.031; MTurk dummy: *β* = 0.182, *p* = 0.004).^22^

The introduction of punishment opportunities strongly increases average contributions in both samples (average contributions in periods 10 vs 11: laboratory: 4.63 vs 12.94, *p* = 0.001; online: 7.85 vs 16.15, *p* = 0.001). Moreover, average cooperation levels over the course of the game are higher than in the absence of punishment (average group contributions in periods 11–20 vs 1–10: laboratory: 16.26 vs 8.30, *p* = 0.001; online: 17.15 vs 12.52, *p* = 0.001). As in the condition without punishment, overall contributions within groups were slightly yet significantly higher in our online sample than in the laboratory (16.26 vs 17.15, *p* = 0.008).^23^

The regression models in Table 2 confirm that MTurk participants initially contribute more to the public good than their laboratory counterparts (Wald test on ‘Constant’: *p* < 0.001; Table 2, columns 1 and 2). Over the course of the game cooperation decays at comparable rates (Wald test on ‘Period’: *p* = 0.624; columns 1 and 2). In the punishment condition, the constant differs between the two samples (Wald test on ‘Constant’: *p* = 0.084; Table 2, columns 4 and 5), but the effect of period is equivalent (Wald test on ‘Period’: *p* = 0.407; Table 2, columns 4 and 5).^24^

**Table 2:** Cooperation dynamics

	Contributions to the public good					
	No punishment			Punishment		
	Laboratory	MTurk	Pooled	Laboratory	MTurk	Pooled
Period	−0.900***	−1.074***	−1.037***	1.139	0.514*	0.682**
	(0.309)	(0.187)	(0.160)	(0.710)	(0.289)	(0.282)
Final period	−3.400	−2.292**	−2.512***	−10.203**	−4.184**	−5.795***
	(2.253)	(0.958)	(0.881)	(4.881)	(1.688)	(1.797)
MTurk			5.421***			4.193
			(1.867)			(4.904)
Constant	10.470***	17.046***	11.402***	25.980***	35.272***	29.601***
	(1.592)	(0.624)	(1.650)	(3.898)	(3.792)	(4.232)
N	720	2480	3200	720	2480	3200
F	8.75	33.66	34.45	2.19	3.12	3.75

Tobit estimation with left-censoring for ‘No punishment’ and right-censoring for ‘Punishment’. ‘Period’ is period number; ‘Final period’ is a dummy for last period; ‘MTurk’ is a dummy for the MTurk sample. Robust standard errors clustered on groups

* *p* < 0.1; ** *p* < 0.05; *** *p* < 0.01

Individual responses to the contributions of fellow group members were significant and similar across the online and laboratory samples (Wald test on ‘Mean peer contribution in *t*-*1*’: *p* = 0.505; Table 3, columns 1 and 2), which suggests that individual decision making online was not more ‘random’ than in the laboratory.^25^

**Table 3:** Cooperation dynamics (no punishment)

	Contribution to the public good (no punishment)		
	Laboratory	MTurk	Pooled
Period	−0.401**	−0.503***	−0.485***
	(0.204)	(0.094)	(0.085)
Final period	−2.826	−1.316	−1.600**
	(1.941)	(0.827)	(0.757)
Mean peer contribution in *t*−1	0.953***	1.043***	1.027***
	(0.125)	(0.060)	(0.054)
MTurk			0.759
			(0.778)
Constant	−0.830	−0.006	−0.696
	(1.674)	(1.237)	(0.931)
N	648	2232	2880
F	29.05	163.74	177.16

Left-censored Tobit estimation. ‘Period’ is period number; ‘Mean peer contribution in *t*−1′ is the average contribution of the other members in the group in *t*−1; ‘MTurk’ is a dummy for the MTurk sample. Robust standard errors clustered on groups

* *p* < 0.1; ** *p* < 0.05; *** *p* < 0.01

### Punishment behavior

**Fig. 3:**
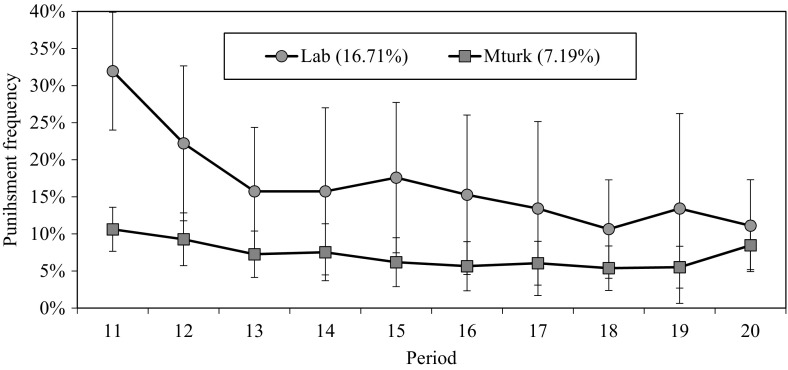
Frequencies of punishment over time. Frequencies are calculated by counting instances of assigning non-zero deduction points out of the total number of punishment opportunities per participant, per recipient, per period. Mean punishment frequencies in parenthesis. *Error bars* indicate 95% confidence intervals clustered on groups

Participants in the online sample punished less often than their laboratory counterparts (overall punishment frequency: 0.072 vs 0.167, *p* = 0.001). Moreover, Fig. 3 shows that in both samples the frequency of punishment tended to decrease over the course of the game, albeit less markedly in our online sample. Accordingly, mean efficiency in the experimental condition with punishment was higher on MTurk than in the laboratory (averages 26.91 vs 22.64 points per individual per period, *p* = 0.002). In cases where participants decided to punish, they did so equally severely in both samples. The average number of points assigned when punishing did not differ significantly between MTurk and the laboratory (4.15 vs 3.88; *p* = 0.545).

**Fig. 4:**
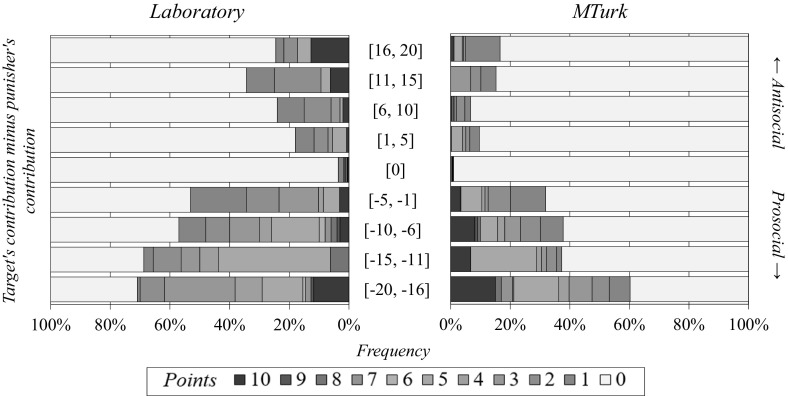
Directionality and severity of punishment in our laboratory and online samples. *Stacked bars* show frequency distributions of punishment decisions. *Each bar* shows the distribution for a given difference between punishers and their target’s contribution to the public good

Figure 4 reveals that, in both samples, punishment was predominantly pro-social in nature: most instances of punishment represented cooperators (who contributed relatively many points to the public good) punishing defectors (who contributed relatively fewer points). In the laboratory and online, both frequency and severity of punishment were higher with increasing differences in contributions between the punisher and their target (Fig. 4, compare the bottom four stacked bars; see Table 8 of the Appendix for regression analyses). Interestingly, we observe some instances of anti-social punishment in both samples (Fig. 4, top four stacked bars).

Table 4 presents an econometric analysis of punishment behavior. It confirms the observations from Figs. 3 and 4. The overall patterns of punishment are similar in the laboratory and online. This analysis further reveals that online participants tended to punish less frequently and less severely, even after controlling for relative contributions and previously received punishment (Table 4, Wald test on constants; columns 1 and 2: *p* = 0.014; columns 3 and 4: *p* = 0.064).^26^

**Table 4:** Determinants of punishment

	Decision to punish (0 = no; 1 = yes)			Punishment severity		
	Laboratory	MTurk	Pooled	Laboratory	MTurk	Pooled
Target’s contribution	−0.181***	−0.216***	−0.203***	−0.505***	−0.717***	−0.641***
	(0.034)	(0.015)	(0.016)	(0.074)	(0.051)	(0.043)
Punisher’s contribution	−0.014	0.003	−0.002	−0.067	−0.011	−0.032
	(0.034)	(0.026)	(0.022)	(0.106)	(0.074)	(0.063)
Mean contrib. others	0.040	0.065**	0.058***	0.136	0.228***	0.197***
	(0.028)	(0.025)	(0.019)	(0.094)	(0.071)	(0.058)
Rec. punishment in *t*−1	0.090***	0.097**	0.092***	0.310***	0.273**	0.284***
	(0.022)	(0.045)	(0.026)	(0.074)	(0.128)	(0.069)
Period	−0.126**	−0.102***	−0.111***	−0.311**	−0.280**	−0.289***
	(0.056)	(0.037)	(0.031)	(0.143)	(0.110)	(0.088)
Final period	−0.633*	0.524*	0.150	−1.014	2.401***	1.271
	(0.336)	(0.294)	(0.266)	(1.220)	(0.888)	(0.780)
MTurk			−0.965***			−2.631***
			(0.231)			(0.803)
Constant	0.960*	−0.440	0.671**	1.569	−1.873*	1.088
	(0.496)	(0.290)	(0.310)	(1.533)	(1.105)	(1.008)
N	2160	7440	9600	2160	7440	9600
Pseudo R^2^	0.285	0.321	0.322	0.142	0.203	0.189

Values in columns 1–3 reflect estimates from logistic models fitted to the decisions to punish (0: no deduction points assigned; 1: at least one deduction point assigned). Values in columns 3–6 reflect effect estimates from left-censored Tobit models fitted to the number of deduction points assigned. ‘Target’s contribution’ is the contribution of the punished participant; ‘Punisher’s contribution’ is the contribution of the participant punishing; ‘Average contribution others’ is the mean contribution of the other two members of the group; ‘Received punishment in t−1′ is the punishment amount received from others in the previous period; ‘Period’ is the period number; ‘Final period’ is a dummy for the last period; ‘MTurk’ is a dummy for the MTurk sample. Robust standard errors clustered on group

* *p* < 0.10; ** *p* < 0.05; *** *p* < 0.01

In sum, our results show that basic patterns of cooperation and punishment behavior in the laboratory are largely replicable online, and thus are robust to changes in the experimental method. Participants in our online sample initially contribute more but, in the absence of punishment opportunities, cooperation decays at similar rates. In both samples, peer punishment is mainly pro-social in nature, and its introduction increases and stabilizes cooperation.

## Attrition: endogenous or exogenous?

Our observation that, across conditions, experimental results are quite robust already suggests that endogenous attrition due to what has happened in the experiment (and hence selection) is not a big issue in our data. Here, we investigate this issue more rigorously.

Crucially, we find no evidence that attrition was *selective* in our experiment: dropout rates did not vary with the experimental condition (absence or presence of punishment). Table 5 details the results of a proportional hazards model (Jenkins 1995) fitted to instances where participants dropped out. The first model confirms the visual impression from Fig. 1 that dropout rates are relatively high in the first period of each experimental condition (columns 1–3). Most likely, this effect is due to the fact that participants have to wait for their group to form (before the start of the first condition), or for all members of their group to finish reading the instructions and completing the comprehension questions (before the start of the second condition). Over the course of each of the conditions, the attrition rates slightly decrease (‘period’ has a negative estimate), suggesting that over time participants become more loyal to the task.

**Table 5:** Determinants of attrition

	Participant’s drop out in period t (0 = no; 1 = yes)				
	Pooled data			Without punishment	With punishment
	(1)	(2)	(3)	(4)	(5)
Punishment available	0.056	0.362	0.107		
	(0.598)	(0.612)	(0.611)		
Period	−0.093*	−0.118**	−0.094*	−0.193***	−0.184**
	(0.051)	(0.053)	(0.053)	(0.066)	(0.077)
First period	2.484***	2.375***	2.554***		
	(0.377)	(0.376)	(0.382)		
Earnings		−0.002	0.011		
		(0.143)	(0.143)		
Group member(s) dropped out in previous period			1.896***	1.677***	2.024***
			(0.382)	(0.500)	(0.574)
Relative average contribution				0.002	−0.082
				(0.025)	(0.053)
Relative average punishment received					−0.105
					(0.086)
Relative average punishment given					−0.100
					(0.086)
Constant	−4.064***	−3.979***	−4.220***	−3.619***	−2.321*
	(0.317)	(0.318)	(0.328)	(0.353)	(1.191)
N	8334	8332	8332	3998	3907
AIC	893.56	875.23	860.27	454.61	303.68

Values reflect estimates from proportional hazards models fitted to binary events of participants staying (0) or dropping out (1) in a given round of the session, conditional on not having dropped out yet. ‘Punishment available’ is a dummy for the presence or absence of punishment; ‘Period’ is the period number; ‘First period’ is a dummy for the first period; ‘Earnings’ reflect participants’ total earnings relative to all other participants in the experiment in a given period; ‘Group member(s) dropped out in previous period’ is a dummy taking the value of 0 (1) when none (at least one) of the group members had left the session in the previous round (potentially delaying the progress within the session); ‘Relative average contribution’ is the participant’s average contribution to the public good minus the average contribution of their fellow group members in all rounds of the session so far; ‘Relative average received (given) punishment’ are the average punishment received (given) by a participant minus the average punishment received (given) by their fellow group members in all rounds so far

* *p* < 0.10; ** *p* < 0.05; *** *p* < 0.01

Models 3–5 show that attrition is much more likely when a group member has dropped out in the previous period. This seems indicative of ‘cascading inattention’: when a participant drops out of the session (e.g. due to inactivity, a closed connection, or waning attention), their group members will have to wait for some time before they can proceed.^27^ Reduced attention may lead to additional attrition.

The results in columns 4 and 5 of Table 5 indicate that attrition did not depend on cooperation and punishment behavior in each of the experimental conditions. Specifically, dropouts did not depend on the behavior of the dropped-out participants relative to their fellow group mates, or on their earnings. The model in column 4 (fitted to the data from the experiment without punishment) shows that dropouts did not depend on relative average contributions. In addition to that, the model in column 5 (fitted to the data from the experiment with punishment) reveals that individuals who dropped out had neither received more punishment relative to their group members who did not drop out, nor differed from them in terms of the punishment towards others.

## Discussion

In light of the results presented here, one might feel tempted to embrace interactive online experimentation as a valuable complement to laboratory studies—and others might even see it as a cost-efficient substitute. The measures presented here address the most important methodological issues for conducting interactive experiments online, and our case study illustrates that established results from the laboratory can be replicated online. However, future research needs to establish how generalizable our results are to other research questions as, despite these measures, methodological differences between laboratory and online experimentation inevitably remain.

For instance, depending on the nature of the experiment, online participants can conceivably communicate with each other to share their knowledge, strategies and even experimental materials more quickly than their laboratory counterparts. As mentioned earlier, most of the forums that monitor the online community have mechanisms in place that prohibit the dissemination of materials, and participants themselves might find this practice prohibitively costly. Yet, one cannot completely rule out this possibility as laboratory and online participants can simply discuss an experiment through other channels. To some extent, the nature of interactive designs prevents participants from crafting intricate strategies beforehand, but this might not be the case for experimental designs where participants can figure out “correct” answers, and they might be at risk of being ineffective (Haigh 2016), or exhibiting reduced effect sizes (Chandler et al. 2015).

Comparisons between online and laboratory experiments can also be affected by differences in selection bias. Participants in online and laboratory experiments may self-select based on their opportunity cost of working time and their reservation wage. Indeed, opportunity costs and reservation wages might well differ between sessions conducted in the laboratory and online: laboratory participants might decide on whether to participate in an experiment by looking at the show-up fee paid and the travel costs they would incur (e.g. walk a long distance, experience bad weather, or even get dressed!), whereas for online participants such costs would typically be negligible. Interestingly though, results from Anderson et al. (2013) show that a comparable type of selection is unlikely to bias inference about the prevalence of other-regarding preferences. Thus, we have reasons to believe that our design is not particularly affected by the relatively low opportunity costs, but that others might be.

In this study, we systematically controlled for what we think are the most daunting logistical issues for running an interactive experiment online. However, one could argue that some important methodological differences between laboratory and online experiments remain, and that such discrepancies may potentially affect findings and treatment comparisons, regardless of experimental designs being interactive or not. For instance, we replicate classic patterns of behavior in an environment with less control but also find an important disparity between initial contributions. As we pointed out earlier though, this divergence is consistent with the one found in related studies comparing different adult populations with college students using various recruitment methods (Belot et al. 2010; Carpenter and Seki 2011; Gächter et al. 2004; Stoop et al. 2012; Gächter and Herrmann 2011). Yet we acknowledge that unobserved methodological differences might account for some of the variability observed in our results. Assessing the extent and severity of such differences in other designs is certainly a topic for future research.

The similarities between our online and laboratory results suggest that interactive designs conducted over the internet can be robust to changes in the experimental method. Yet, our results are based on a comparison between sessions that differed in terms of both the experimental method (online versus in the physical laboratory) and the subject pool (MTurk workers versus university participants). Further assessment of online experimentation could include a systematic study of the isolated effects of the method for collecting data (online or laboratory) on the one hand, and the subject pool (‘workers’ from an online labor market or university students) on the other hand, e.g. by running online experiments with university students and inviting MTurkers into the physical laboratory.

To summarize our discussion, we see our paper as a guide for researchers to think about relevant issues before deciding whether the online or the physical laboratory is most appropriate for their research question. Some might conclude that the loss of control is too big a problem for their designs, whereas others are willing to bear that loss of control. In the end, the extent of any loss of control is an empirical question and we encourage researchers to add to our first piece of experimental evidence.

## Summary and conclusion

In this paper, we presented a detailed conceptual and methodological discussion of conducting interactive experiments in the physical laboratory and online. We illustrated similarities and differences using a repeated public goods experiment without and with punishment. Our comparative results suggest that online data quality is adequate and reliable, making online interactive experimentation a potentially valuable complement to laboratory studies.

Most importantly, attrition, though a significant nuisance in online experiments, did not compromise the internal validity of our data because attrition was unrelated to what happened in our experiment. Future research will need to establish how generalizable this result is to other interactive decision problems, in particular when attrition might be treatment-specific, which poses the biggest problem to internal validity (Zhou and Fishbach 2016). Future research should also investigate how individual characteristics of participants (e.g. social preferences) and aspects of the experimental design (e.g. group size, number of periods, complexity of the task and its instructions) affect dropouts.

We observed that cooperation levels in our online sample are substantially higher than in the laboratory, and are on the high end of the range of cooperation levels observed in the cross-cultural samples of Herrmann et al. (2008). These differences can be partly (but not completely) explained by the age of MTurkers relative to students in typical laboratory samples. Still, it is unclear whether some other differences in terms of the participants’ demographics, the perceived degree of anonymity, or the degree of familiarity with the experimental paradigm influence our results. We believe that future research should explore such avenues.

### Electronic supplementary material

Below is the link to the electronic supplementary materials.

Supplementary material 1 Instructions and additional figures (PDF 4521 kb)

Supplementary materials 2 Experimental data and analysis files (Zip 14,352 kb)

## Appendix—additional statistical analyses

See Tables 6, 7 and 8.

**Table 6:** Cooperation dynamics

	Contribution to the public good							
	Tobit estimation				Multilevel mixed effects estimation			
	No punishment (left censored)		Punishment (right censored)		No punishment		Punishment	
Period	−1.037***	−1.042***	0.682**	0.711**	−0.731***	−0.737***	0.091***	0.093***
	(0.160)	(0.159)	(0.282)	(0.281)	(0.043)	(0.044)	(0.024)	(0.025)
Final period	−2.512***	−2.611***	−5.795***	−5.864***	−1.277***	−1.315***	−1.119***	−1.082***
	(0.881)	(0.907)	(1.797)	(1.863)	(0.408)	(0.420)	(0.233)	(0.241)
MTurk	5.421***	5.390**	4.193	8.282	4.218***	5.084***	0.893	2.009
	(1.867)	(2.130)	(4.904)	(5.919)	(1.394)	(1.466)	(1.249)	(1.320)
Age		0.120**		−0.003		0.028		−0.029
		(0.058)		(0.144)		(0.029)		(0.025)
Female		2.370**		−4.072		0.927*		0.118
		(0.976)		(2.595)		(0.516)		(0.438)
Single child		0.730		−1.096		0.051		−0.245
		(1.262)		(4.071)		(0.715)		(0.607)
Foreign		−1.042		6.286		0.801		0.401
		(2.557)		(4.622)		(1.196)		(1.065)
Membership		1.242		5.536		1.764***		1.520***
		(1.452)		(4.661)		(0.651)		(0.556)
Constant	11.402***	6.544**	29.601***	26.270***	12.451***	10.043***	15.871***	15.368***
	(1.650)	(2.778)	(4.232)	(6.756)	(1.246)	(1.530)	(1.109)	(1.348)
N	3200	3050	3200	3050	3200	3050	3200	3050
Chi^2^/F	34.45	15.35	3.75	1.96	503.80	496.65	25.67	32.03

Tobit and Multilevel mixed effects estimation, which allows for individual and group differences, as well as for treatment-specific residuals. ‘Period’ is period number; ‘Final period’ is a dummy for last period; ‘MTurk’ is a dummy for the MTurk sample; ‘Age’ is the participant’s age; ‘Female’ is a dummy for female participants; ‘Foreign’ is a dummy for participants who grew up outside the US; ‘Membership’ is a dummy for participants who were members of a social club. Robust standard errors clustered on groups for the Tobit model

* *p* < 0.1; ** *p* < 0.05; *** *p* < 0.01

**Table 7:** Cooperation dynamics (no punishment)

	Contribution to the public good (no punishment)			
	Tobit estimation		Multilevel mixed effects estimation	
Period	−0.485***	−0.492***	−0.500***	−0.499***
	(0.085)	(0.090)	(0.052)	(0.054)
Final period	−1.600**	−1.741**	−0.812**	−0.864**
	(0.757)	(0.781)	(0.394)	(0.407)
Mean peer contribution in* t*−1	1.027***	1.000***	0.461***	0.460***
	(0.054)	(0.058)	(0.027)	(0.027)
MTurk	0.759	1.827	2.252***	2.980***
	(0.778)	(1.142)	(0.862)	(0.985)
Age		0.033		0.036
		(0.039)		(0.031)
Female		2.022***		1.230**
		(0.674)		(0.557)
Single child		0.128		0.128
		(1.017)		(0.789)
Foreign		−0.321		0.191
		(1.903)		(1.315)
Membership		2.153**		1.692**
		(0.975)		(0.690)
Constant	−0.696	−3.582**	7.073***	4.412***
	(0.931)	(1.599)	(0.870)	(1.296)
N	2880	2745	2880	2745
Chi^2^/F	177.16	80.17	822.66	799.84

Left-censored Tobit and multilevel mixed effects estimation, which allows for individual and group differences, as well as for treatment-specific residuals. ‘Period’ is period number; ‘Final period’ is a dummy for the last period; ‘Mean peer contribution in *t*−1′ is the average contribution of the other members in the group in *t*−1; ‘MTurk’ is a dummy for the MTurk sample; demographic controls are the same of Table 6. Robust standard errors clustered on groups; Robust standard errors clustered on groups for the Tobit model

* *p* < 0.1; ** *p* < 0.05; *** *p* < 0.01

**Table 8:** Determinants of peer punishment

	Decision to punish (0 = no; 1 = yes)							
	Logit estimation							
	Prosocial punishment				Antisocial punishment			
	Lab	MTurk	Pooled	Pooled and controls	Lab	MTurk	Pooled	Pooled and controls
Punisher’s contribution	0.046	−0.003	0.011	−0.002	−0.143***	−0.153***	−0.142***	−0.130***
	(0.051)	(0.023)	(0.022)	(0.026)	(0.024)	(0.026)	(0.018)	(0.020)
Target’s contribution	−0.059	−0.118***	−0.100***	−0.097***	−0.013	0.010	0.002	0.004
	(0.038)	(0.029)	(0.023)	(0.023)	(0.025)	(0.021)	(0.015)	(0.020)
Others’ avg. contribution	0.038	0.054***	0.047***	0.065***	−0.025	−0.019	−0.023	−0.017
	(0.035)	(0.019)	(0.017)	(0.017)	(0.028)	(0.024)	(0.018)	(0.019)
Received *p* in* t*−1	−0.034	−0.025	−0.028	−0.076**	0.129***	0.069**	0.094***	0.103***
	(0.027)	(0.045)	(0.023)	(0.032)	(0.031)	(0.034)	(0.023)	(0.025)
Period	−0.117***	−0.111**	−0.113***	−0.103***	0.024	0.036	0.031	0.032
	(0.039)	(0.044)	(0.032)	(0.032)	(0.062)	(0.056)	(0.041)	(0.048)
Final period	−0.115	−0.077	−0.077	−0.236	−1.800***	0.595*	−0.159	−0.014
	(0.341)	(0.366)	(0.275)	(0.313)	(0.587)	(0.340)	(0.388)	(0.434)
MTurk			−0.855***	−0.983***			−1.132***	−1.983***
			(0.307)	(0.302)			(0.347)	(0.403)
Age				0.005				0.025
				(0.017)				(0.019)
Female				−1.171***				−0.078
				(0.260)				(0.381)
Single child				−0.320				0.099
				(0.376)				(0.457)
Foreign				1.403*				−2.380***
				(0.739)				(0.628)
Membership				0.019				−0.838*
				(0.288)				(0.462)
Constant	0.206	0.202	0.854*	1.216*	−0.560	−2.137***	−0.878**	−1.101
	(0.812)	(0.393)	(0.466)	(0.712)	(0.379)	(0.682)	(0.442)	(0.682)
N	370	900	1270	1201	1790	6540	8330	7949
Chi^2^	27.51	38.98	48.60	109.88	111.55	85.36	187.39	254.26

	Decision to punish (0 = no; 1 = yes)							
	Multilevel mixed effects estimation							
	Prosocial punishment				Antisocial punishment			
	Lab	MTurk	Pooled	Pooled and controls	Lab	MTurk	Pooled	Pooled and controls
Punisher’s contribution	0.000	0.003	0.002	0.000	−0.008***	−0.004***	−0.005***	−0.004***
	(0.006)	(0.005)	(0.004)	(0.004)	(0.001)	(0.001)	(0.000)	(0.000)
Target’s contribution	−0.012***	−0.021***	−0.018***	−0.018***	0.000	0.000	0.000	0.000
	(0.004)	(0.003)	(0.002)	(0.002)	(0.002)	(0.001)	(0.001)	(0.001)
Others’ avg. contribution	0.013***	0.011***	0.012***	0.015***	0.000	−0.001	−0.001	0.000
	(0.005)	(0.003)	(0.003)	(0.003)	(0.001)	(0.001)	(0.001)	(0.001)
Received *p* in* t*−1	−0.009	−0.004	−0.007	−0.012***	0.010***	0.003***	0.004***	0.004***
	(0.005)	(0.006)	(0.004)	(0.004)	(0.001)	(0.001)	(0.001)	(0.001)
Period	−0.014	−0.011**	−0.012***	−0.013***	0.004*	0.001	0.001	0.001
	(0.009)	(0.005)	(0.004)	(0.004)	(0.002)	(0.001)	(0.001)	(0.001)
Final period	−0.037	−0.045	−0.043	−0.040	−0.081***	0.018***	0.010*	0.011*
	(0.086)	(0.047)	(0.041)	(0.042)	(0.019)	(0.006)	(0.006)	(0.006)
MTurk			−0.190***	−0.215***			−0.044***	−0.088***
			(0.059)	(0.072)			(0.016)	(0.018)
Age				0.001				0.001
				(0.003)				(0.001)
Female				−0.204***				0.001
				(0.052)				(0.011)
Single child				−0.085				0.002
				(0.076)				(0.016)
Foreign				0.154				−0.071**
				(0.105)				(0.028)
Membership				−0.070				−0.035**
				(0.063)				(0.014)
Constant	0.611***	0.437***	0.622***	0.700***	0.187***	0.104***	0.156***	0.176***
	(0.123)	(0.092)	(0.084)	(0.122)	(0.042)	(0.016)	(0.020)	(0.027)
N	370	900	1270	1201	1790	6540	8330	7949
Chi^2^	24.64	98.78	129.04	168.92	138.89	116.77	197.44	226.82

Logit and Multilevel mixed effects estimation, which allows for individual and group differences, as well as for treatment-specific residuals. We split the analysis into two different types of punishment. Pro-social punishment includes instances where the punisher’s contribution to the public good in that round exceeded that of their target. Anti-social punishment includes instances where the target contributed at least as much as the punisher. ‘Punisher’s contribution’ is the contribution of the participant punishing; ‘Target’s contribution’ is the contribution of the punished participant; ‘Average contribution others’ is the mean contribution of the other two members of the group; ‘Received punishment in* t*−1′ is the punishment amount received from others in the previous period; ‘Period’ is the period number; ‘Final period’ is a dummy for the last period; ‘MTurk’ is a dummy for the MTurk sample; demographic controls are the same of Table 6. Robust standard errors clustered on groups for the Logit model

* *p* < 0.10; ** *p* < 0.05; *** *p* < 0.01

## Footnotes

1 MTurk is by no means the only online labor market used for behavioral research. Peer et al. (2017) identified six other platforms for crowdsourcing behavioral research, in addition to platforms such as *Prolific Academic*. See Buhrmester et al. (2011), Horton et al. (2011), Rand (2012), and Paolacci and Chandler (2014) for evaluations of the potential of MTurk for conducting behavioral experiments in psychology and other social sciences.

2 For example, Chesney et al. (2009) conduct classic experiments in a virtual world platform and observe behaviour similar to the laboratory. Hergueux and Jacquemet (2015) find that social preferences of student participants elicited online and offline are qualitatively very similar; see their Table 1 for further references. Schmelz and Ziegelmeyer (2015) compare the behaviour of student participants in a principal-agent experiment conducted in the laboratory and on the Internet and conclude that data can be of similar quality. On MTurk, Horton et al. (2011) replicate classic framing effects and report similar levels of cooperation in a one-shot prisoner’s dilemma played online and in the laboratory. Berinsky et al. (2012) replicate classic findings on framing and risk tasks. For overviews of replication studies, see Paolacci et al. (2010), Behrend et al. (2011), Klein et al. (2014), and Mullinix et al. (2015).

3 Thus far interactive research developed its own bespoke software (see Egas and Riedl 2008; Suri and Watts 2011; Wang et al. 2012; Gallo and Yan 2015; Nishi et al. 2015; Schmelz and Ziegelmeyer 2015; Stagnaro et al. 2017), but recent software is likely to change this trend (see BreadBoard, MobLab, oTree, SoPHIE, and UbiquityLab).

4 In one of the first studies using economic games on MTurk, Suri and Watts (2011) replicate the laboratory results found by Fehr and Gachter (2000) using a repeated public goods game without punishment.

5 Another early paper on experimentation on the internet is Reips (2000). The author discusses the advantages and disadvantages of conducting online experiments and also provides a methodological discussion of how internet experiments can be used to validate laboratory data.

6 Using four canonical one-shot games, Amir et al. (2012) find results comparable to those found in the laboratory, even when using relatively low stakes. In the public goods context, Suri and Watts (2011) also report similar levels of cooperation when using two different compensation levels.

7 In comparison to the student or community samples normally used in the laboratory, MTurk samples are also more diverse (Krupnikov and Levine 2014; Paolacci and Chandler 2014; Weinberg et al. 2014; Berinsky et al. 2012).

8 Also, the size of the potential pool of participants on MTurk allows researchers to use designs requiring large groups, and to obtain sufficient statistical power to detect effects of relatively small size (e.g. Hauser et al. 2016).

9 We chose a 20-min time slot based on pilot sessions. Over time, MTurk HITs move down the list of most recently-published jobs and entrance rates tend to taper off. When participants trickle in at low rates, the chances that they have to wait for a long time before they can be matched into a group increase substantially.

10 Self-selection into experiments may not be a specific problem for online studies. Selection effects may also occur in physical laboratories which regularly run interactive tasks. Participants may anticipate their tasks involving interactions with other participants in their session due to previous experience or hearing about it from other members of the subject pool; see Krawczyk (2011), Anderson et al. (2013), Cleave et al. (2013) and Abeler and Nosenzo (2015) for detailed discussions.

11 Stewart et al. (2015) estimate a worker half-life of about seven months and Rand et al. (2014) report a median of 300 academic studies, 20 of which occurred in the past week.

12 We used *UniqueTurker* (http://uniqueturker.myleott.com) to prevent re-takes between sessions. This method uses a unique MTurk identification number which is linked to each worker and that Amazon constantly monitors to avoid duplicate participation. Alternatives for these methods include *Turkprime* (http://turkprime.com).

13 We used considerably shorter experimental instructions than Herrmann et al. (2008) to minimize the variation in the time that participants take to read them; with dynamic group formation, minimal variation in preparation time is highly desirable (e.g. to reduce attrition, see below).

14 When entering an incorrect answer, subjects were allowed to try again. Incidentally, the total number of incorrect attempts in the six comprehension questions prior to Condition 1 did not differ between our laboratory and online samples (4.68 vs 4.46, respectively, Mann–Whitney test: *p* = 0.245).

15 As subjects completed the experiment in their browsers, we strictly regulated navigation between experimental pages. Participants navigating the pages at will may lead to serious disruptions in the experimental flow and even lead to crashing sessions. We used LIONESS to regulate the sequence of an experiment in a browser by using a pop-up window without navigation bars and overwriting the browser history within that window when moving on to another page. This procedure also prevented participants from revising their decisions during a given interaction.

16 On waiting pages (to which LIONESS directed participants once they made their decisions and waited for their fellow group members to do so as well), we added an animation (a commonly used circular icon spinning around) to assure the participants that the experiment was still active. Additional measures to promote attention may include playing sounds (e.g. Hauser et al. 2016) or having the browser window with the experimental pages placed in the foreground of the participants’ computer screen (‘focus stealing’) when a decision is due. We did not use these more intrusive measures in this study.

17 The experimental instructions mentioned that the total number of points contributed to the public good was multiplied by 1.6 before distributing the resulting amount equally among the group members. A group member dropping out therefore increased the marginal per capita return of contributions.

18 Note that both the stability of the experimental software and the stability of the server on which the software is run might strongly affect attrition too. Pilot sessions led us to deploy LIONESS on high-CPU servers (available through Google Cloud) to facilitate many simultaneous server–client interactions.

19 Participants that did not respond in time or dropped out for any other reason did not receive any payment.

20 We incurred additional costs in our online sessions through: (1) 60 participants who did complete the comprehension questions but were not allocated to a group and who received $1; (2) participants from groups with dropouts (a total of $725.54); (3) Amazon MTurk fees ($489.02). The online costs per usable data point is computed as: (payments to the participants in the 62 groups finishing without dropouts + additional costs from 1–3)/62. This amount does not include payments to subjects who took part in pilots we ran to determine our procedures.

21 Unless otherwise stated, all statistical tests are two-sided Mann–Whitney tests with group averages over all periods as independent observations.

22 See List (2004) for a detailed discussion of age effects on cooperation behavior. These results are also consistent with Gächter and Herrmann (2011) who found in a laboratory one-shot public goods game that older participants contribute more to the public good than younger ones.

23 See Online Appendix B for a comparison of our samples with the cross-cultural ones from Herrmann et al. (2008).

24 An additional regression model including an interaction between ‘MTurk’ and ‘period’ does not detect a significant effect of this interaction in either condition (*p* > 0.560). Our results are also robust to the inclusion of demographic controls and the use of a different (multilevel mixed effects) model specification (see Table 6 of the Appendix).

25 An additional model with an interaction between ‘MTurk’ and ‘mean contributions’ shows no significance for this item (*p* = 0.294). As in Table 2, results are robust to different model specifications (see Table 7 of the Appendix).

26 In Table 8 of the Appendix we present a more detailed analysis of pro-social and anti-social punishment, indicating that by-and-large, punishment is driven by the same factors in the laboratory and online. These models also reveal that both in the laboratory and online the positive effects of ‘received punishment in *t*−1′ on punishment reported in Table 4 are due to instances of anti-social punishment.

27 In our software a subject was considered to have dropped out after 20 s of inactivity. This delay was introduced in order to allow people with brief connection irregularities to still complete the experiment.
