# Supplementary material for: Conducting interactive experiments online
Source: Exp Econ. 2017 May 9;21(1):99–131. doi: 10.1007/s10683-017-9527-2 (PMC5807491; doi:10.1007/s10683-017-9527-2)
Supplement: Supplementary file 1 — Supplementary material 1 Instructions and additional figures (PDF 4521 kb) [file 10683_2017_9527_MOESM1_ESM.pdf]

# Online Appendix to “Conducting interactive experiments online”

**Antonio A. Arechar · Simon Gächter · Lucas Molleman**

## Online Appendix A – Experimental Instructions

Below is the description of the HIT of our MTurk experiment, as seen by participants.

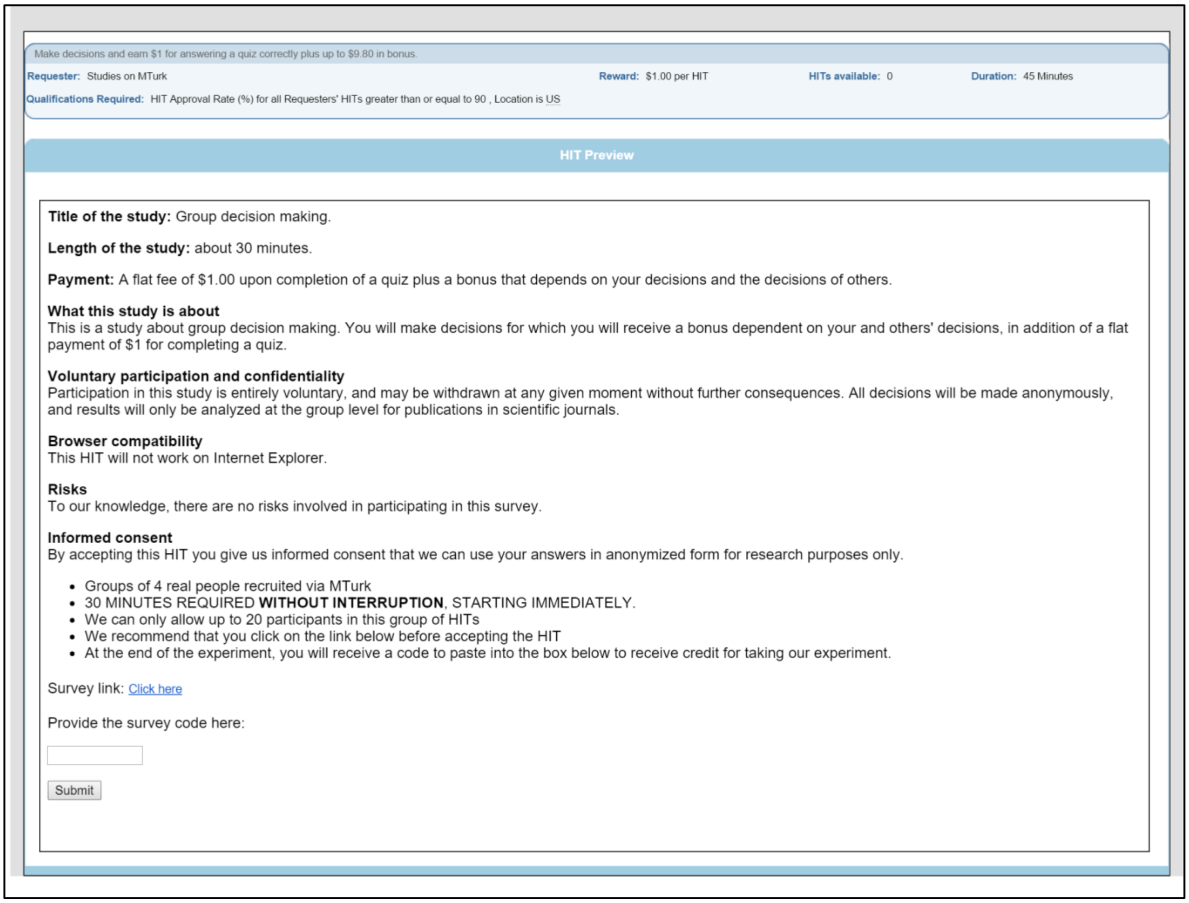

Make decisions and earn $1 for answering a quiz correctly plus up to $9.80 in bonus.

**Requester:** Studies on MTurk **Reward:** $1.00 per HIT **HITs available:** 0 **Duration:** 45 Minutes

**Qualifications Required:** HIT Approval Rate (%) for all Requesters' HITs greater than or equal to 90 , Location is US

**HIT Preview**

**Title of the study:** Group decision making.

**Length of the study:** about 30 minutes.

**Payment:** A flat fee of $1.00 upon completion of a quiz plus a bonus that depends on your decisions and the decisions of others.

**What this study is about**
This is a study about group decision making. You will make decisions for which you will receive a bonus dependent on your and others' decisions, in addition of a flat payment of $1 for completing a quiz.

**Voluntary participation and confidentiality**
Participation in this study is entirely voluntary, and may be withdrawn at any given moment without further consequences. All decisions will be made anonymously, and results will only be analyzed at the group level for publications in scientific journals.

**Browser compatibility**
This HIT will not work on Internet Explorer.

**Risks**
To our knowledge, there are no risks involved in participating in this survey.

**Informed consent**
By accepting this HIT you give us informed consent that we can use your answers in anonymized form for research purposes only.

- Groups of 4 real people recruited via MTurk
- 30 MINUTES REQUIRED **WITHOUT INTERRUPTION**, STARTING IMMEDIATELY.
- We can only allow up to 20 participants in this group of HITs
- We recommend that you click on the link below before accepting the HIT
- At the end of the experiment, you will receive a code to paste into the box below to receive credit for taking our experiment.

Survey link: Click here

Provide the survey code here:

Submit

Moreover, below are the instructions given to all of our participants (laboratory and MTurk). Each box corresponds to the screens shown.

## Instructions

In this experiment you will play a game with the same three people for 10 rounds. In these rounds, you can earn Points. At the end of the experiment these Points will be converted into real money **(50 Points = 1 Dollar)**.

### Your task

At the beginning of each round, each participant receives 20 Points. You have to decide how many of the 20 Points you want to **contribute to a group project**. The other three members of your group make this decision at the same time.

The Points you do not contribute, you **keep for yourself**. These Points are added to your total. After all group members have made their decision, all Points contributed to the **group project** are added up, and this number of Points is **multiplied by 1.6**. The resulting number of Points is then divided equally among the group members (irrespective of how much they individually contributed to the group project).

**In summary**:
Your income in a round =
The Points you keep for yourself
*plus*
The Points you receive from the group project

**Group project - Example 1**

- All four players contribute 20 Points to the group project.
- Sum of contributions is 80 Points.

- This amount is multiplied by 1.6, resulting in 128 Points.
- Each participant receives (128/4=) 32 Points from the group project.

- Therefore, the income of each player is 32 Points.

**Group project - Example 2**

- Participants A, B and C contribute each 20 Points to the group project.
- Participant D contributes 0 Points.
- Sum of contributions is 60 Points.
- This amount is multiplied by 1.6, resulting in 96 Points.
- Each participant receives (96/4=) 24 Points from the group project.

- Therefore, the income of Participants A, B and C is 24 Points.
- The income of Participant D is 44 Points (20 kept for himself *plus* 24 from the group project).

Please make your decision within the **time limit** shown on your screen.

After all members of your group have made their decision, the results of the round will be shown to you. Once all players in your group are finished, a new round will begin. Again, you will receive 20 Points to start with. After 10 rounds, this first experiment is over. A second and final experiment will follow. Further information about this second experiment will be provided later.

Please click the link below if you understood the instructions.

Before the experiment itself starts, a brief quiz will check whether you understand your task.

[I have read and understood the instructions. Continue!]

## Quiz

Please answer all control questions. They serve as a test for your understanding of the experiment.

1. At the start of a round, each group member receives 20 Points. Suppose nobody (including you) contributes any Points to the group project. What is:
a) Your income?
b) The income of each of the other group members?

2. At the start of a round, each group member receives 20 Points. Suppose you contribute 20 Points to the group project. All other group members each contribute 20 Points to the group project. What is:
a) Your income?
b) The income of each of the other group members?

3. At the start of a round, each group member receives 20 Points. Suppose the other three group members contribute a total of 40 Points to the group project.
a) What is your income if you contribute 0 Points to the group project?
b) What is your income if you contribute 10 Points to the project?

[Submit]

The first experiment of this session is now over. A second and final experiment will follow.

## Instructions for the second experiment

In this second experiment you will again play a game in the **same group** for **10 rounds**. In each round your task is **very similar** to your task in the first experiment, but we introduce **one change**. After all group members have made their contributions to the group project, you can **assign Deduction Points** to each of the other members of your group.

For each Deduction Point you assign, **3** Points will be deducted from the total of the recipient, and **1** Point will be deducted from your total. In each round, you can assign **between 0 and 10 Deduction Points** to each of the other members of your group.

**Deduction Points - Example**

- All members of your group have made their contribution to the group project.
- As before, your screen displays the results of this round so far.
- You assign the following Deduction Points to Participants A, B and C: 2, 0 and 3.
- This reduces your income in this round by (2+0+3=) 5 Points.

- The other Participants assign a total of 4 Deduction Points to you.
- This reduces your income in this round by (4x3=) 12 Points.

- In this round, your total income reduction due to Deduction Points is (5+12=) 17 Points.

Please be aware that when many Deduction Points are assigned to you, your income in a round may become **negative**. In that case, your income in that round will be set to 0 (zero). However, you will **always** incur the costs of assigning Deduction Points to others.

After all members of your group have assigned their Deduction Points, the results of the round will be shown to you. Once all players in your group are finished, a new round will begin. After 10 rounds of this game, a brief questionnaire will follow concluding this session. Your payment for this session will consist of:

Your guaranteed $10 participation fee
+ Your income from the 10 rounds of the first experiment
+ Your income from the 10 rounds of the upcoming second experiment

At the start of this second experiment, you will receive **an extra 25 Points** to compensate for potential losses due to Deduction Points.

Please click the link below if you understood the instructions. A brief quiz will check whether you understand your task before the experiment itself starts.

[I have read and understood the instructions. Continue!]

## Quiz

Please answer all control questions. They serve as a test for your understanding of the experiment.

1. All members of your group have made their contributions to the group project. Suppose that you assign the following Deduction Points to the other members of your group: 2, 3 and 0. By many Points will your income be reduced by assigning Deduction Points in this round?

2. By many Points will your income be reduced if you assign 0 Deduction Points in total?

3. By how many Points will your income be reduced if you receive a total of 7 Deduction Points from the other members of your group?

[Submit]

[Go back to instructions of the upcoming experiment]

## Online Appendix B – Cooperation and punishment on MTurk from a wider perspective

Although aggregate dynamics of cooperation were similar across our samples, cooperation levels were appreciably higher online than in the laboratory, especially in the absence of punishment (Figure 2 of the main text). Indeed, in comparison to the wide spectrum of cooperation levels observed in a large cross-cultural sample (Herrmann et al. 2008), our online sample ranks at the very top (Figures B1 and B2). Specifically, in the absence of punishment our online sample tops the ranks (Fig. B1). In the presence of punishment it starts with the highest cooperation levels and ranks third in average cooperation (Fig. B2), while punishment is used less frequently than in any other sample (Fig. B3).

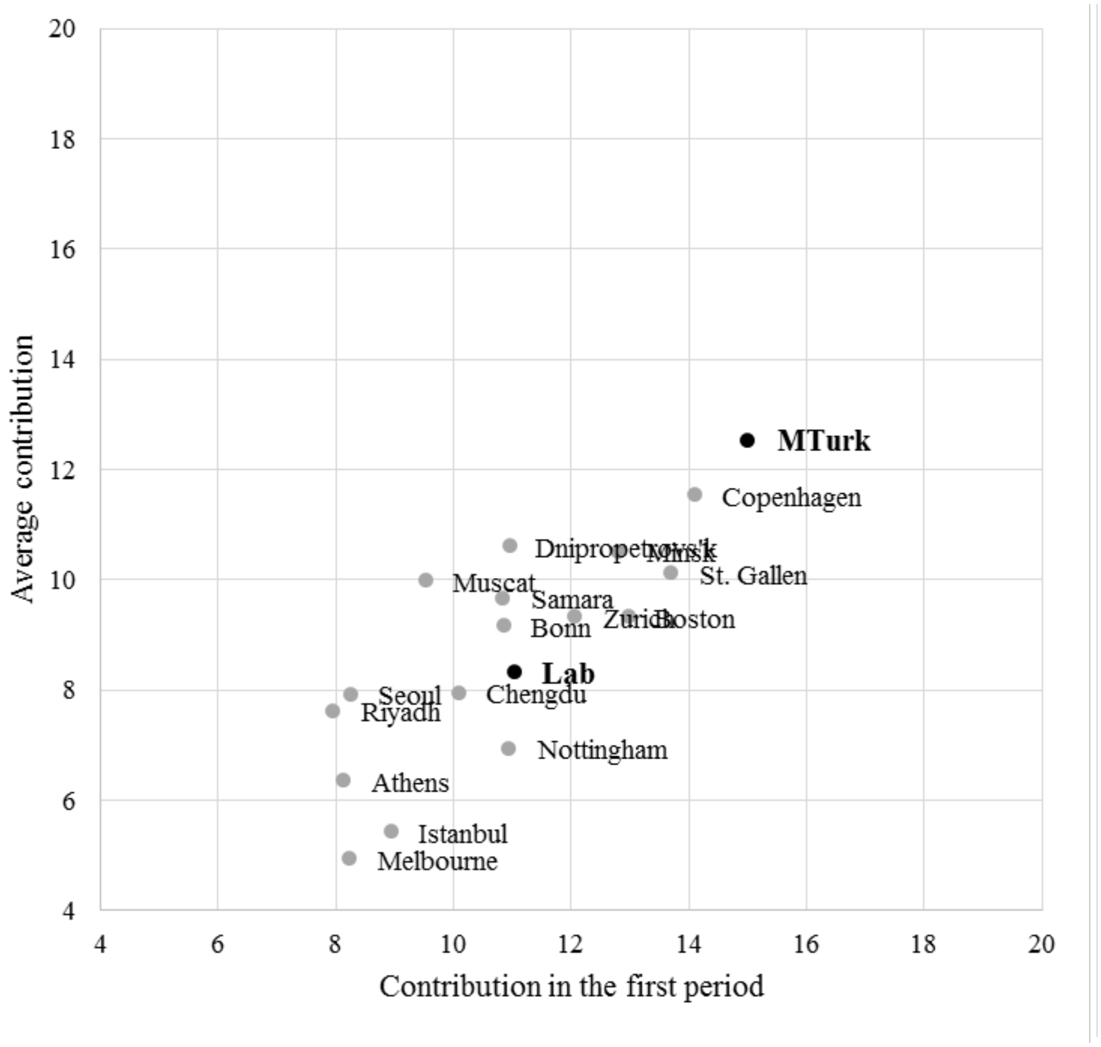


**Fig. B1** Contributions in the first period and average contribution in the No-Punishment condition in our samples from MTurk, the laboratory, and the 16 cities included in Herrmann et al. (2008)

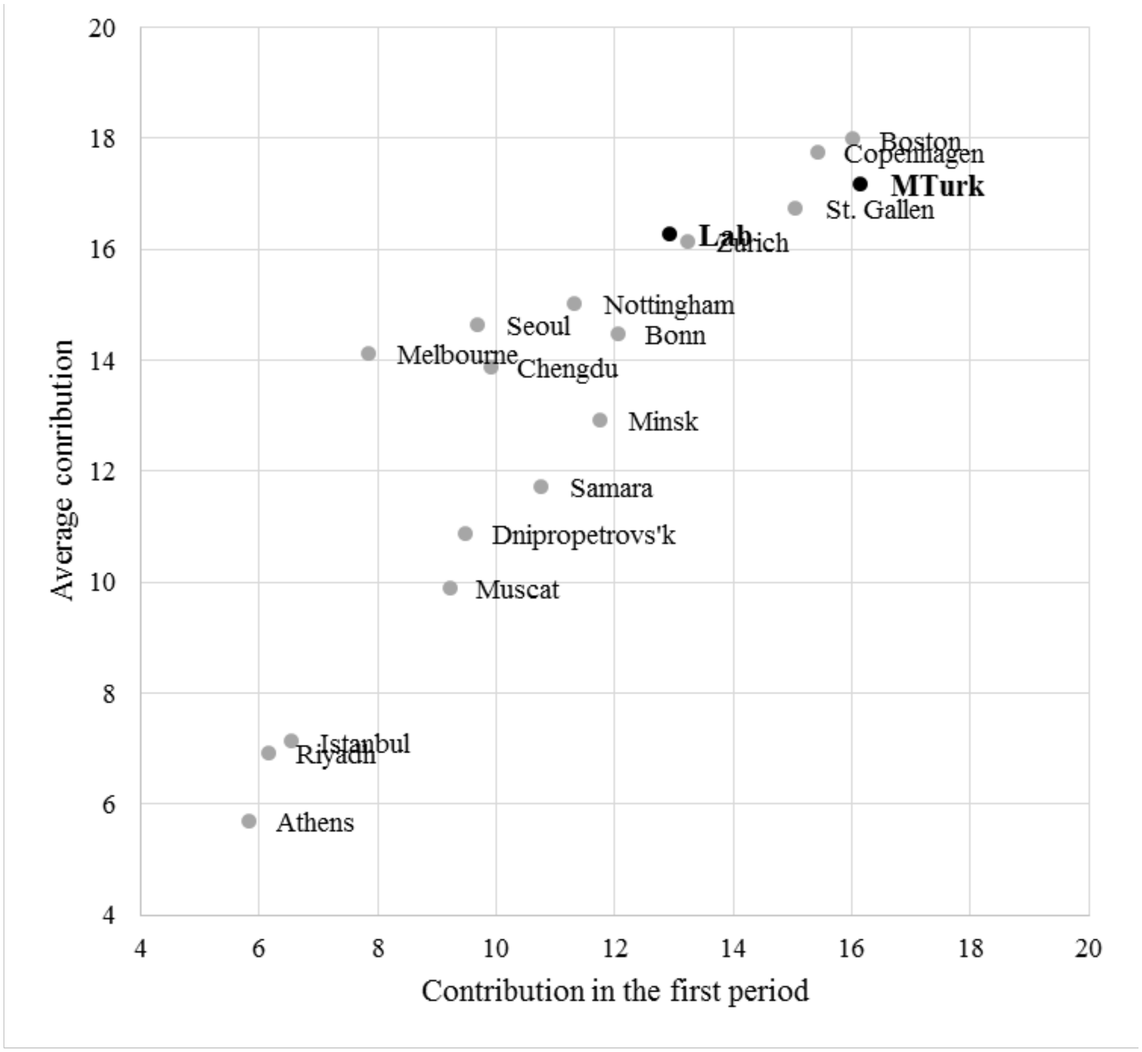


**Fig. B2** Contributions in the first period and average contribution in the Punishment condition in our samples from MTurk, the laboratory, and the 16 cities included in Herrmann et al. (2008)

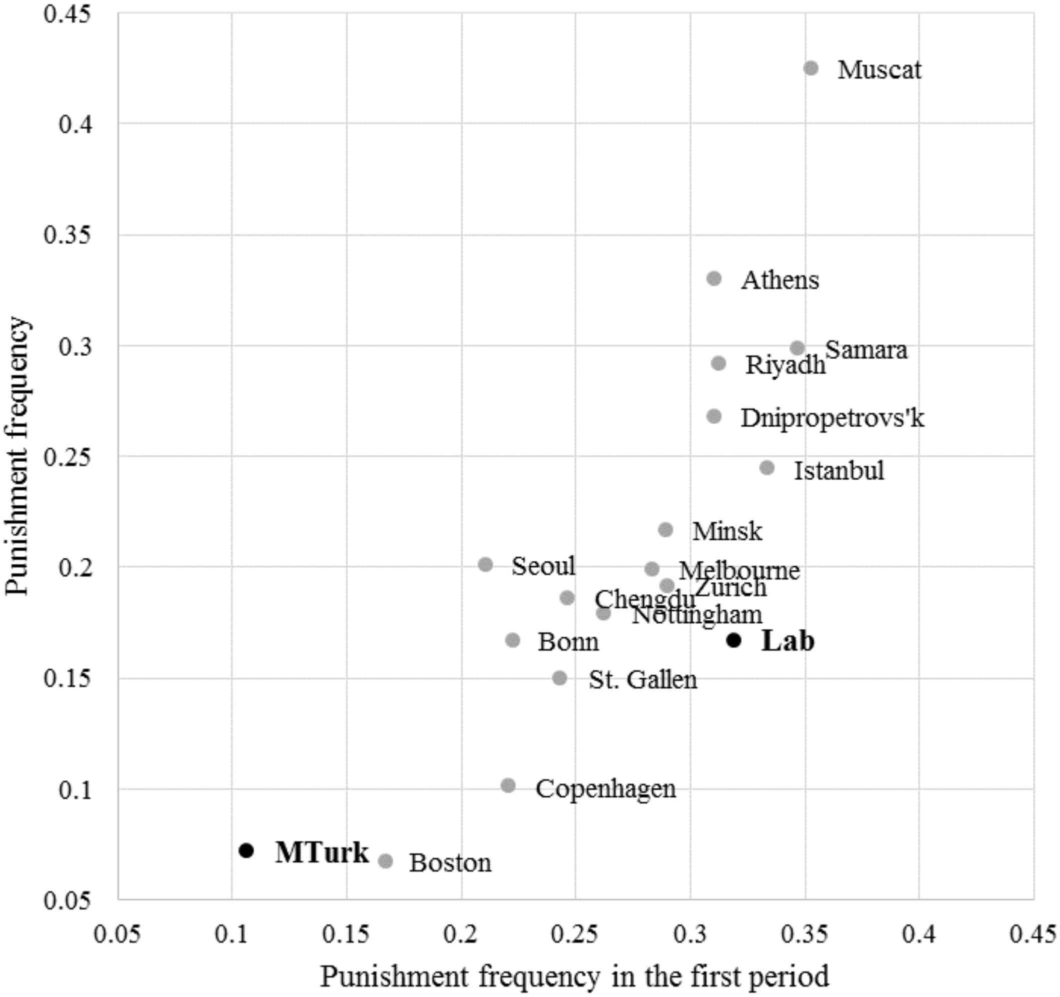


**Fig. B3** Punishment frequencies in the first period and overall frequencies in our samples from MTurk, the laboratory, and the 16 cities included in Herrmann et al. (2008)
